# Individual and community-level determinants of Iron-Folic Acid Intake for the recommended period among pregnant women in Ethiopia: A multilevel analysis

**DOI:** 10.1016/j.heliyon.2021.e07521

**Published:** 2021-07-09

**Authors:** Abay Woday Tadesse, Setognal Birara Aychiluhm, Kusse Urmale Mare

**Affiliations:** a Samara University, College of Medicine and Health Sciences, Department of Public Health, Samara, Ethiopia; b Armauer Hansen Research Institute, Addis Ababa, Ethiopia; c Dream Science and Technology College, Amhara region, Dessie, Ethiopia; d Samara University, College of Medicine and Health Sciences, Department of Nursing, Samara, Ethiopia

**Keywords:** Pregnant women, Iron-Folic Acid intake, Multilevel analysis, Ethiopia

## Abstract

**Background:**

Iron-folic acid (IFA) intake for the recommended period during pregnancy reduces the risk of anemia and congenital anomalies. However, IFA intake for the recommended period is still very low in low-income countries including Ethiopia. Thus, the aim of this study was to assess both individual-and community-level determinants of IFA intake for the recommended period among pregnant women in Ethiopia.

**Methods:**

Data were retrieved from the Demographic and Health Survey program's official database website (http://dhsprogram.com). A two-stage stratified cluster sampling technique was employed to conduct the 2016 Ethiopian Demographic and Health Survey. A sample of 3088 pregnant women who had received at least one dose of IFA in Ethiopia were included in this study. A multivariable multilevel logistic regression analysis model was fitted to identify the determinants of IFA intake below the recommended period [< 90 days] during pregnancy. Akaike's Information Criterion (AIC) was used during the model selection procedure.

**Results:**

This study revealed that 87.6% [95% CI; 86.3%, 88.6%] of the women took IFA below the recommended period during the index pregnancy. After adjusting for the covariates: living in rural areas [AOR = 1.74: 95% CI 1.37, 2.50], and women's illiterate proportion [AOR = 1.43: 95% CI 1.06, 1.70] were community level factors. Whereas, primary education level [AOR = 0.63: 95% CI 0.40, 0.78], poorer wealth index [AOR = 1.53: 95% CI 1.08, 3.09], 4 + antenatal care visits [AOR = 0.43: 95% CI 0.31, 0.69], and receive nutritional counseling during pregnancy [AOR = 0.63: 95% CI 0.37, 0.84] were the individual-level factors of IFA intake below the recommended period during pregnancy.

**Conclusions:**

In this study, nearly nine out of ten pregnant women did not take IFA for the recommended period. Thus, promoting recommended ANC visits, enhancing the quality of nutritional counseling, strengthening the expansion of media, and educate rural women towards the importance of optimal intake of IFA during pregnancy. Besides, the policymakers should design essential strategies based on identified barriers to improve the IFA intake for the recommended period.

## 1 Introduction

Iron folic acid intake among pregnant women is a core process indicator of the global nutrition monitoring framework [1, 2] that helps to halt the burden of iron deficiency anemia worldwide, especially in low-income countries like Ethiopia [3]. Iron with folic acid is an important micronutrient for physiological function, growth, and development as well as maintenance of life for the mother and her fetus during pregnancy and in later life [2, 3, 4, 5]. Optimal adherence to iron-folic acid supplementation during pregnancy reduced the risk of anemia related morbidities for the mother, and congenital anomalies like neural tube defects for the fetus [6, 7, 8, 9]. On the other hand, the intake of IFA below the recommended period during pregnancy has adverse neonatal outcomes such as; miscarriage, stillbirth, prematurity, low birth weight, congenital anomalies, perinatal morbidity, and mortality [10, 11, 12]. Consequently, Iron-folic acid supplementation for the recommended period is currently a crucial strategy to prevent adverse birth outcomes and hematologic complications during pregnancy [3, 5, 8, 13, 14, 15].

The Demographic and Health Survey report across twenty-two developing countries showed that 81% of all pregnant women had received IFA tablets. Of those receiving IFA tablets, only 8% consumed 180 or more IFA tablets [16]. In Sub-Saharan African countries, the overall prevalence of adherence to ≥90 days of iron supplementation during pregnancy was 28.7% [17]. In Ethiopia, two-third of pregnant women had at least one antenatal care visit but only 42% of women took IFA supplementation during pregnancy [18].

Previous studies conducted in different settings have been identified different factors associated with IFA intake for the WHO recommended period i.e. 90 days or more. The identified factors include; maternal socioeconomic status and sociodemographic factors [17, 19, 20], lack of recommended ANC visits [17, 21], intolerance of gastrointestinal side effects while taking the tablets [22], inadequate supply of IFA tablets/syrups and poor counseling towards IFA utilization [19, 23], inadequate utilization of prenatal health-care services [21], and poor family/relative support [19, 24].

Currently, various strategies and interventions have been implemented to improve maternal and child health at global and national levels [25, 26, 27]. In Ethiopia, iron-folic acid supplementation is the main strategy to prevent maternal death due to anemia and adverse birth outcomes [28, 29, 30]. However, IFA intake for the recommended period of 90 days or more remains very low in the country.

Moreover, limited studies were conducted in Ethiopia where the primary focus of the studies was to assess whether the pregnant women were supplemented with IFA or not [21, 23, 31, 32]. However, these studies were not accounted for the IFA intake for the recommended period or not among pregnant women. Besides, the previous studies conducted in the country were not identified the community-level factors of IFA intake for the recommended period. Because a single-level logistic regression analysis of a hierarchical nature of data violates the independence assumptions of regression [33, 34]. Therefore, this study was intended to address both the individual- and community-level factors of IFA intake below the recommended period among pregnant women in Ethiopia using mixed models.

## 2 Methods and materials

### 2.1 Study area and data source

The data were retrieved from the Demographic and Health Survey (DHS) program's official database website (http://dhsprogram.com).

The Ethiopian demographic and health survey (EDHS-2016) was conducted in nine regions [Tigray, Afar, Amhara, Oromia, Somali, Benishangul-Gumuz, Southern Nations Nationalities and Peoples Region (SNNPR), Gambella, and Harari), and two cities administrative (Addis Ababa and Dire-Dawa)]. This survey was conducted from January 18, 2016, to June 27, 2016.

The EDHS-2016 was conducted based on a two-stage stratified cluster sampling technique. Enumeration areas (EAs) were selected in the first stage. In the second stage, 28 households per EA were selected with an equal probability of systematic selection per cluster. Nationally, a total of 645 EAs were selected with probability proportional to EA size. In this survey, a sample of 16,515 women aged 15–49 years was collected.

A weighted 3088 pregnant women who had received IFA supplements and who were asked how many days they consumed IFA tablets/syrup during their last pregnancy that occurred four years prior to the national survey were included [18] and the dataset we used is only maternal dataset.

### 2.2 Study variables

**Dependent Variable:** The outcome variable of this study was IFA consumption for the recommended period, i.e. 90 days or more [17, 35] which was dichotomized into “IFA consumption for less than 90 days = 1, IFA consumption for 90 or more days = 0”.

**Independent Variables:** All the independent variables were classified into individual-level and community-level variables.

*Individual-level variables*: maternal age, age at first marriage, religion, wealth index, women's and her husband's occupation and education level, family size, parity, gravidity, mass media exposure, and ANC visits.

*Community-level variables:* Community-level women's illiteracy proportion, community-level media exposure, distance from health facilities, region, place of residence, and cluster. Community-level women's education and women's media exposure proportions were not directly found in the DHS data but they might be a significant factor for the outcome variable. Thus, these two variables were created by aggregating the individual-level factors (women's no education and no mass media exposure). Besides, these variables were dichotomized as community-level women's low and high illiteracy proportion using the median. Because the proportion of illiterate level among women was not normally distributed. To measure exposure to mass media in the 2016 EDHS, watching television (TV), listening to radio, and reading newspapers at least once a week were considered. Therefore, a new variable “media exposure at individual-level” was generated by combining the three media sources (TV, Radio, and Newspaper), Then, media exposure was labeled as “Yes” if respondents had exposure to at least one of the media channel and labeled “No” if respondents did not have any exposure to either of the three media channels. Finally, similar to community-level women's illiteracy proportion, we created the aggregated data and categorized the community-level no mass media exposure proportion into low and high no media exposure using the median (50%).

In this study, the region was recoded into three categories: peripheral regions (Afar, Somali, Benshangul-gumz, and Gambella), Central regions (Amhara, Tigray, SNNP, and Oromia), and Metropolis regions (Addis Ababa, Dire Dawa, and Harar) [36].

### 2.3 Data analysis

#### 2.3.1 Descriptive statistics

The data were weighted using primary sampling unit to restore the representativeness of the data and to get a reliable estimate. Then, the proportions and frequencies were estimated after applying the weighted sample for disproportionate sampling and non-responses. This was done based on the detailed explanation of the weighting procedure of the EDHS sample weighting technique [37]. Categorization and re-categorizations were done for continuous variables using information obtained from different works of literature. The analysis was performed using Stata version 15.0.

#### 2.3.2 Multivariable multilevel logistic regression analysis

In this study, due to the hierarchical nature of the 2016 EDHS data (i.e. pregnant women are nested within clusters), to account for this clustering effect, a multivariable multilevel logistic regression analysis was used to estimate the effects of individual and community-level determinants of IFA consumption for the recommended period among pregnant women.

The eligible independent variables for the final model were screened using different techniques. Therefore, individual and community-level variables with a p-value of less than 0.25, the absence of collinearity between independent variables, and clinical importance of the independent variables were considered to select the candidate predictor variables into the multivariable multilevel logistic regression analysis model.

#### 2.3.3 Model building and selecting the best-fitted model

The four models (Model I-IV) containing variables of interest were fitted using Stata version 15.0 to check and select the best-fitted model. 1) Model I, “called the null model, was fitted without explanatory variables to test random variability in the intercept and to estimate the intra-class correlation coefficient (ICC), and Proportion Change in Variance (PCV). 2) Model II was fitted to examine the effects of individual-level variables on the outcome interest. 3) Model III *was also fitted to* examine the effect of community-level predictors on the outcome of interest. 4) The final Model IV, called the Full model, was fitted to examine the effects of both individual and community-level independent variables on the outcome of interest simultaneously.

Akaike's Information Criterion (AIC) was applied to compare the four models and then the model with low AIC value was considered as the best-fitted model. Therefore, the full model was the best-fitted model for this data (Table 2). AOR with 95% Confidence interval in the multivariable multilevel logistic regression model was used to select the independent predictors of IFA consumption below 90 days among women.

### 2.4 Ethical approval

Not applicable but we have obtained letter of permission to access the dataset for analysis from demographic and health survey program through the online request at: http://www.dhsprogram.com.

## 3 Results

### 3.1 Descriptive statistics of the study variables

This study involved 3088 women who were taking Iron and Folic Acid five years preceding the survey. The median age of the participants was 28 years with 9 Interquartile range (IQR). Out of the total participants; 2528 (81.9%) were living in rural areas, 1879 (61.4%) were gotten first marriage before 18 years of age, 1665 (53.9%) were not attended formal education, 1494 (48.4%) were unemployed, and 503 (16.3%) had poorest wealth index. In this study, 2778 (89.9%) had ANC follow-ups during the index pregnancy, and 1782 (57.7%) had no media exposure towards the IFA intake for the recommended period. The study has also shown that 1515 (49.1%) were not accessible to nearby health facilities (Table 1).

**Table 1:** Weighted socio-demographic characteristics of study participants [weighted N = 3088].

List of predictor variables	Category of variables	Frequency	Percent
Place of residence	Urban	560	18.1
	Rural	2528	81.9
Religion	Orthodox	1498	48.5
	Protestant	605	19.6
	Muslim	919	29.8
	Others +	66	2.1
Age at first marriage	<18 years	1879	61.4
	18 + years	1182	38.6
Respondent's educational status	No education	1665	53.9
	Primary	1002	32.4
	Secondary	264	8.6
	Higher	157	5.1
Husband's educational status	No education	1232	42.3
	Primary	1125	38.7
	Secondary	322	11.1
	Higher	230	7.9
Respondent's occupation	Unemployed	1494	48.4
	Employed	1594	51.6
Husband's occupation	Unemployed	173	5.9
	Employed	2736	94.1
Wealth Index	Poorest	503	16.3
	Poorer	655	21.2
	Middle	607	19.7
	Richer	636	20.6
	Richest	687	22.3
Family size	<5	1096	35.5
	5+	1992	64.5
Head of the household	Males	2651	85.9
	Females	437	14.1
Media Exposure	Yes	1306	42.3
	No	1782	57.7
Number of ANC visits	1–3	1227	49.8
	4+	1551	50.2
Total children ever born (gravidity)	1–4	2102	68.1
	5+	986	31.9
Number of live births (parity)	<5	2266	73.4
	5+	822	26.6
Get nutritional counsel during pregnancy	Yes	2042	73.5
	No	736	26.5
Distance to health facilities	Big problem	1515	49.1
	Not big problem	1573	50.9
Proportion of community-level women's illiteracy	Low	1213	39.3
	High	1875	60.7
Proportion of community-level no media exposure	Low	1425	46.2
	High	1663	53.8
Region of the country	Peripheral regions	150	4.8
	Large central	2788	90.3
	Metropolis	150	4.9

Key: Others+ (Catholic, Traditional, and other EDHS categories).

### 3.2 Iron-Folic Acid Intake for the recommended period among pregnant women

This study revealed that 2703 (87.6%) [95% CI; 86.3%, 88.6%] women had IFA intake below the recommended period (less than 90 days). In this study, 1,939 (72.5%) women who were living in rural areas and 1,389 (53.9%) of the non-educated women were not taking IFA for the recommended period.

### 3.3 Determinants IFA intake among pregnant women

#### 3.3.1 Null model

In the null model, the variance of the random factor was 1.32 (95% CI 1.09, 1.88), showing heterogeneous areas. The variance estimate is greater than zero that indicates a significant difference in enumeration areas (clusters) where pregnant women's iron intake below the recommended period is found. The ICC indicated that 29% of the total variability in iron intake below the recommended period is due to differences across cluster areas, in which the remaining unexplained 71% is attributed to the individual differences. Besides, the median odds ratio that was 2.97 in the null model indicates that there was a variation of iron supplementation between clusters. Thus, a multilevel analysis was considered as an appropriate approach for further analysis. The PCV indicated that 64.4% of the variation in iron intake below the recommended period across communities was explained by both individual-and community-level factors included in the full model (Table 2).

**Table 2:** Community- and individual-level variance of two-level mixed-effect logit models predicting IFA intake below the recommended period among pregnant women in Ethiopia.

Random effect	Null model	Full model
Community-level variance	1.32	0.47
ICC (%)	29	22
Median odds ratio (MOR)	2.97	1.78
PCV (%)	Reference	64.4
Model fitness statistics using AIC	2187	1892

#### 3.3.2 Full model

In the multilevel multivariable logistic regression model, both the individual and community level factors were fitted simultaneously. Thus, place of residence, women's illiteracy level at the community, women's education level, poorer wealth index, number of ANC visits, and nutritional counseling during pregnancy were statistically associated with IFA intake below recommended period at 95% confidence level.

This study revealed that pregnant women in rural residence had 74% higher odds of IFA intake below recommended compared to those living in an urban area [AOR = 1.74: 95% CI 1.37, 2.50]. This study indicated that women's high illiterate proportion at the community level increased the odds of IFA intake below recommended by 43% compared to their counterparts [AOR = 1.43: 95% CI 1.06, 1.70]. In this study, women who attended primary education level were 37% less likely to have IFA intake below the recommended period compared to illiterate women (AOR = 0.63: 95% CI 0.40, 0.78). The odds of IFA intake below recommended period among pregnant women in the households with a poorer wealth index was 53% greater compared to their counterparts (AOR = 1.53: 95% CI 1.08, 3.09). In this study, women who had four or more ANC visits were 57% less likely to have IFA intake below the recommended period compared to women who had below four ANC visits (AOR = 0.43: 95% CI 0.31, 0.69). Finally, women who received nutritional counseling during pregnancy had 37% fewer odds of IFA intake below the recommended period compared to those women who did not have any counseling during pregnancy (AOR = 0.63: 95% CI 0.37, 0.84). However, distance from health facilities, sex of the household head, media exposure, family size, husband's education level, maternal age, region, and community-level media exposure were not statistically associated with below recommended IFA intake among pregnant women (Table 3).

**Table 3:** Multivariable multilevel logistic regression analysis of factors associated with IFA intake below recommended among pregnant women in Ethiopia, EDHS 2016.

List of variables	Category of variables	Model II (community-level) AOR with 95% CI	Model III (Individual-level) AOR with 95% CI	Model IV (Full model) AOR with 95% CI
Residence	Urban	1.00	–	1.00
	Rural	1.27 (1.09, 1.82)∗	–	1.74 (1.37, 2.50)∗
Proportion of No media exposure at community	Low	1.00	–	1.00
	High	1.23 (0.81, 1.84)	–	0.94 (0.57, 1.54)
Proportion of illiteracy level	Low	1.00	–	1.00
	High	1.52 (1.09, 2.36)∗	–	1.43 (1.06,1.70)∗
Region category in the country	Peripheral	1.00	–	1.00
	Central	1.25 (0.83, 1.86)	–	1.38 (0.88,2.17)
	Metropolis	0.56 (0.33, 0.94)	–	0.71 (0.40,1.25)
Age category	15–24	–	1.00	1.00
	25–34	–	1.21 (0.75, 1.95)	1.21 (0.44, 1.95)
	35+	–	0.87 (0.51, 1.47)	0.87 (0.51, 1.49)
Respondent's educational status	Illiterate	–	1.00	1.00
	Primary	–	0.63 (0.41, 0.86)∗	0.63 (0.40, 0.78)∗
	Secondary	–	0.90 (0.45, 1.80)	0.83 (0.42, 1.85)
	Higher	–	0.60 (0.23, 1.57)	0.53 (0.22, 1.57)
Husband's educational status	Illiterate	–	1.00	1.00
	Primary	–	0.76 (0.49, 1.19)	0.73 (0.48, 1.20)
	Secondary	–	1.19 (0.64, 2.22)	1.13 (0.63, 2.26)
	Higher	–	0.59 (0.27, 1.26)	0.58 (0.27, 1.25)
Wealth Index	Poorest	–	1.00	1.00
	Poorer	–	1.63 (1.08, 3.14)∗	1.53 (1.08, 3.09)∗
	Middle	–	1.62 (0.87, 3.00)	1.53 (0.84, 2.94)
	Richer	–	1.56 (0.80, 3.05)	1.53 (0.76, 2.98)
	Richest	–	1.30 (0.59, 2.83)	1.23 (0.49, 3.12)
Family size	<5	–	1.00	1.00
	5+	–	1.30 (0.88, 1.94)	1.33 (0.87, 1.95)
Head of the household	Males	–	1.00	1.00
	Females	–	0.75 (0.42, 1.35)	0.73 (0.42, 1.40)
Media Exposure	No	–	1.00	1.00
	Yes	–	0.81 (0.54, 1.23)	0.83 (0.52, 1.30)
Number of ANC visits	1–3 visits	–	1.00	1.00
	4 + visits	–	0.46 (0.30, 0.69)∗	0.43 (0.31, 0.69)∗
Get nutritional counsel during pregnancy	Yes	–	0.65 (0.37, 0.85)∗	0.63 (0.37, 0.84)∗
	No	–	1.00	1.00
Distance to health facilities	Big problem	–	1.00	1.00
	Not big problem	–	0.89 (0.57, 1.37)	0.89 (0.59,1.36)

Key note: ∗-p value <0.05, AOR-Adjusted Odds Ratio, CI-Confidence Interval.

## 4 Discussion

This study revealed that a low proportion of pregnant women took IFA for a recommended period although it is highly associated with a one-third reduction of under-five and neonatal mortality [5, 38, 39]. Moreover, this study has identified that residence, number of ANC visits, community-level women's illiteracy proportion, wealth index, women's education, and nutritional counseling during pregnancy as the predictors of IFA intake below the recommended period during pregnancy.

This study revealed that 87.6% of the women had IFA intake below the recommended period. This finding is higher than studies conducted in 22 Sub-Saharan African countries (71.3%) [17], EDHS report (57.9%) [18], Aykel town, Northwest Ethiopia (52.4%) [23], Afar region, northeast Ethiopia (77%) [19], Misha district, South Ethiopia (60.8%) [32], and Khartoum, Sudan (34.6%) [40]. Similarly, the prevalence of low IFA intake in this study is higher than studies conducted in Twenty-two countries (8%) [16], Debre Tabor General Hospital, Ethiopia (66%) [41], Tigray, Ethiopia (59.1%) [42], Eritrean refugee camps, northern Ethiopia (35.3%) [43], evidence from the 2011 EDHS (82.9%) [44], Kiambu County, Kenya (67.3%) [45], North-Western Tanzania (79.7%) [46], and Pakistan (61.7%) [47]. However, this finding is lower than studies conducted in eight rural districts of Ethiopia (96.5%) [48], and Northern Ethiopia (89.5%) [49]. The discrepancies could be justified related to differences in quality of service delivered in the facility, sociocultural barriers, women's compliance for the service, and women's awareness on the importance of IFA intake during pregnancy. Besides, the differences in the access to health care facilities, and availability of IFA in the nearby health institutions might also contribute to these variations.

In this study, the odds of IFA intake below recommended period among pregnant women in a rural residence was 74% higher compared to those living in urban areas. This finding is consistent with studies conducted in the Afar region, northeast Ethiopia [19], Indonesia [50], and Pakistan [47]. This could be justified by the fact that women in rural areas may not be accessible to antenatal health care services [46], information regarding the importance of IFA intake adherence, and other maternal health services compared to women in urban residence. Thus, these women are less likely to take their IFA tablets/syrups for the recommended period than women in urban areas.

This study revealed that pregnant women with a high proportion of illiteracy at the community-level had 43% greater odds of IFA intake below the recommended period compared to women with a low proportion of illiteracy. On the other hand, women who attended primary education level were 37% less likely to have IFA intake below the recommended period compared to illiterate women. This finding is similar to studies conducted in 22 Sub-Saharan African countries [17], Ethiopia [31], evidence from the 2011 EDHS [44], Pakistan [47], a systematic review of 15 studies [51]. This could be due to education is a vital tool to enhance knowledge of pregnant women about the importance of IFA intake during pregnancy [52]. Besides, illiterate women are highly associated with the underutilization of available antenatal care services in the community [50]. Thus, the education level of the women is the main predictor of the IFA intake during pregnancy.

This study indicated that the odds of taking IFA below recommended period among pregnant women with a poorer wealth index was 53% greater compared to their counterparts. This finding is consistent with studies conducted in 22 Sub-Saharan African countries [17], Ethiopia [31], Pakistan [47]. The possible explanation could be due to the reason that women in households with poorer wealth index may not have good access to maternal health care service utilization [35] that could be related to financial constraints for transportation and other maternal services.

In this study, women who had four or more ANC visits were 57% less likely to have IFA intake below the recommended period compared to women who had below four ANC visits. This finding is similar to studies conducted in 22 Sub-Saharan African countries [17], Afar region, northeast Ethiopia [19], Ethiopia [31], and India [35], Khartoum, Sudan [40], eight rural districts of Ethiopia [48], Northern Ethiopia [49], Eritrean refugee camps, northern Ethiopia [43], evidence from the 2011 EDHS [44], Pakistan [47], a systematic review of 15 studies [51]. The possible justification might be mothers who had four or more ANC visits may have adequate information about the importance of IFA intake adherence [46] and may took the prescribed IFA tablets for the recommended period compared to the women who have fewer than four antenatal care visits. In addition, women who had four and above ANC visits may have a positive attitude towards maternal health services including iron use that enables them to take their IFA for the recommended period compared to their counterparts.

This study indicated that women who received nutritional counseling during pregnancy had 37% fewer odds of IFA intake below the recommended period compared to those women who did not have any counseling during pregnancy. This finding is similar to studies conducted in the Afar region, northeast Ethiopia [19], Misha district, south Ethiopia [32], Debre Tabor General Hospital, Ethiopia [41], Tigray, Ethiopia [42], Eritrean refugee camps, northern Ethiopia [43], eight rural districts of Ethiopia [48], Northern Ethiopia [49], and systematic review of 15 studies [51]. Women who received counseling regarding the importance of IFA intake [35] during pregnancy can be more adhered to their prescribed IFA tablets/syrup compared to women who did not receive adequate counseling during their ANC follow-ups.

### 4.1 Strengths and limitations of the study

This study was done based on the EDHS data that have a representative sample size. Moreover, this study applied multilevel modeling to handle the hierarchical nature of the EDHS data. However, the study might have recall bias since the participants were asked about the events happened 5 years or more preceding the survey.

## 5 Conclusions

In this study, nearly nine out of ten pregnant women were not taking their IFA for the recommended period. The multivariable multilevel model showed that rural residence area, four or more ANC visits, the proportion of illiteracy at community-level, poorer wealth index, women education level, and received nutritional counsel during pregnancy were the predictors of IFA intake among pregnant women. Thus, promoting recommended ANC visits, enhancing the quality nutritional counseling, and promoting the awareness of rural women towards the importance of optimal intake of IFA during pregnancy are essential strategies to improve the utilization of iron supplements for the recommended period. Therefore, it enables policymakers to design and prioritize follow-up activities to more precisely identified barriers.

## Declarations

### Author contribution statement

Abay Woday Tadesse, Setognal Birara Aychiluhm and Kusse Urmale Mare: Conceived and designed the experiments; Analyzed and interpreted the data; Wrote the paper.

### Funding statement

This research did not receive any specific grant from funding agencies in the public, commercial, or not-for-profit sectors.

### Data availability statement

Data included in article/supplementary material/referenced in article.

### Declaration of interests statement

The authors declare no conflict of interest.

### Additional information

No additional information is available for this paper.
